# Patient-reported effectiveness and safety of Pamidronate in NSAIDs-refractory chronic recurrent multifocal osteomyelitis in children

**DOI:** 10.1007/s00296-021-04886-4

**Published:** 2021-05-20

**Authors:** Bartłomiej Juszczak, Jerzy Sułko

**Affiliations:** Department of Orthopaedics, Children’s University Hospital of Cracow, ul. Wielicka 265, 30-663, Cracow, Poland

**Keywords:** NSAIDs-refractory chronic recurrent multifocal osteomyelitis, Chronic recurrent multifocal osteomyelitis, Chronic non-bacterial osteitis, Pamidronate, Bisphosponates

## Abstract

To evaluate patient-reported effectiveness, safety and social influence of Pamidronate in the therapy of NSAIDs-refractory Chronic Recurrent Multifocal Osteomyelitis in children. Authors reviewed self-created questionnaires, which asked patients for symptoms alleviation, adverse drug reactions frequency and degree of severity and daily activities self-reliance. Only surveys with complete answers, which were returned to authors by an e-mail from juvenile patients treated for NSAIDs-refractory Chronic Recurrent Multifocal Osteomyelitis at the University Children’s Hospital of Cracow were analyzed. Between 2010 and 2019, 61 children were diagnosed with NSAIDs-refractory Chronic Recurrent Multifocal Osteomyelitis at our department. Out of 61 requests sent, 42 complete replies (33 females, 9 males) were gathered and analyzed. All patients included in this research were administered with at least one set of Pamidronate intravenously in the dose of 1 mg/kg/day for 3 consecutive days. Our analysis shows remarkable in terms of patient’s impressions decrease of pain intensity after 2.5 series of Pamidronate on average, and total pain resolution after 5.9 series on average. Overall number of adverse drug reaction events reported by responders was 105. One patient developed drug-dependent renal insufficiency in the course of therapy. Outcome assessment indicates that nearly 50% of the studied population was more eager to participate in social life just after the first infusion of the drug. 95% of the surveyed unanimously agreed to recommend Pamidronate therapy to cure NSAIDs-refractory CRMO. 39 out of 42 (93%) patients considered Pamidronate effective at the end of the treatment. Onset of Pamidronate’s action is gradual and differs in terms of symptoms alleviation between sexes. The therapy can induce considerable number of adverse drug reactions (2.5 per patient). Only 3 out of 42 (7%) patients were free from any ADRs. To demonstrate the impact of the use of Pamidronate on daily activities more precisely, further research with quantification of the quality of life is warranted.

## Introduction

Chronic Recurrent Multifocal Osteomyelitis (CRMO) is an inflammatory, yet non-infectious disease that presents itself with pain, fever and enlargement of affected bones. It was first reported by Giedon [1] as “symmetrical” lesions of osteomyelitis. Recently, the thinking has shifted towards a more probable cause for the onset of CRMO, abandoning infection as its source, instead putting forward new concept of innate dysregulation between pro-inflammatory (TNF-, Il-20, Il-6) and anti-inflammatory (Il-10, Il-9) factors [2–6]. The Diagnostic scoring system invented by Jansson [7] provides us with a clinically useful tool in distinguishing non-bacterial osteomyelitis from infectious osteomyelitis; thus CRMO has stopped being considered merely as a diagnosis of an exclusion.

Several attempts have been made to establish standards of treatment of CRMO in juvenile group of patients. Notwithstanding the fact that non-steroidal anti-inflammatory drugs (NSAIDs) [8–10] have been recommended as a first-line therapy [11], bisphosphonates have recently gained in importance and are progressively considered as a preferential treatment, especially when the disease is refractory to NSAIDs treatment. [12]. On the other hand, the vicarious use of bisphosphonates is considered to be related to a greater quantity of adverse drug reactions. Additionally, the evidence on bisphosphonates efficacy in CRMO treatment is based on small population groups [13–15], which undermines their efficiency and safety. Taking into account these limitations, the authors of this paper conducted research on a larger group of pediatric patients diagnosed with NSAIDs-refractory CRMO, who were treated with successive infusions of Pamidronate.

The aim of our study was to conduct a survey among the target pediatric group and assess the patient’s-reported effectiveness of Pamidronate, along with any adverse drug effects and further implications on patients’ social life and daily activities.

## Methods

### Survey design

The survey-based study was developed in accordance with the outlines presented in the Journal of Korean Medical Science [16]. Authors used unambiguous and simple language as the target group was a juvenile population. Open-ended inquiries were excluded to provide clear and consistent replies. Insights and feedback from patients were sought to support the design of the questionnaire regarding the prevalence of common side effects in treatment decision-making. Questions on daily activities were based on the CHAQ questionnaire. The survey was internally validated prior to the dissemination with the help of two, independently interviewed patients. Each questionnaire was accompanied with a cover letter providing details of the survey’s purpose, methodology of the research, as well as instruction on how to fill in the form. Considering the young age of participants, it was suggested that the document be filled in with parental supervision. E-mail addresses of the pediatric population used for distribution of the form were collected from patient’s ambulatory medical records.

The survey was developed to obtain information not only about the patient’s perspective on Pamidronate’s effectiveness in alleviating signs and symptoms of CRMO, but also about regaining self-reliance in daily activities. A 4-point scale was used to gage the patient’s view on the level of difficulty involved in performing those activities. Authors hypothesized an improvement in the course of the therapy, so as to have a better insight into the patient’s response to the drug, we divided the time frame into three periods: before drug intake, after first dose intake and after third dose intake. A subsequent part included questions about particular adverse drug reactions, such as episodes of fever, flu-like symptoms, lightheadedness, skin rash, rigors or abdominal pain. Patients were also asked, if any additional, not aforementioned ADRs occurred, as well as if any ADRs persisted longer than for 3 days. A following part of the form comprised questions about Pamidronate influence on the patient’s social life, relationship with peers or frame of mind.

### Study design

This cross-sectional survey was administered to 61 patients under 18 years of age diagnosed with chronic recurrent multifocal osteomyelitis disease (CRMO) refractory to NSAIDs therapy. 42 out of 61 surveys directly sent to the target population were eventually included in this study and analysed by the authors. Each form returned, comprised the identity of the responder. We excluded surveys with incomplete answers or patients older than 18 years old. All pediatric patients were treated under the same conditions at the University Children’s Hospital of Cracow (UCH) by the same doctor between 2010 and 2019. All patients included in this study underwent an initial course of NSAIDs that lasted 4 weeks as indicated by Zhao [1]. In cases with previous history of CRMO therapy, we considered the failure of a shorter, 2-week-long initial trial as an indication to begin second-line therapy with Pamidronate. After first-line treatment failure, all patients were administered with weight-based doses of Pamidronate (bisphosphonate) intravenously for 3 consecutive days. The same drug infusion set was repeated every 6–12 weeks depending on the intensity of the patient’s symptoms. Only patients with at least one set of drug infusion were included in the study. At the time of the survey, children received more than 8 series (2–22 series) on average. In cases with a prolonged onset of Pamidronate action, no other causal treatment was implemented. No additional inclusion or exclusion criteria were applied.

## Results

### Effectiveness of Pamidronate

The study took place from November 2019 to January 2020 in Cracow, Poland. A total of 42 (68.8%) out of 61 e-mail requests sent out were returned. The majority of responders were females 33 (78.5%) with a mean age of 13.9 (7–18). The remaining group comprised 9 males (21.5%) with a mean age of 14.8 (9–18). 27 females (81.8%) and 6 males (66.7%) had already finished therapy, while 6 females and 3 males were still continuing the Pamidronate therapy. The most common dosage regimen between each series of drug infusion was 2 months (35.7%). At the time of filling in the survey, males had been administered with a mean of 5.8 series of Pamidronate, while females with 8.8 series. The median duration of the use of pamidronate was 66 weeks. The contemporary drug intake characteristic is presented in Table 1.

**Table 1:** Contemporary drug intake (at the moment of filling out the survey)

Finished	Unfinished
*Females = 33 (100%)*	
0–5 series—3 (9%)	0–5 series—3 (9%)
6–10 series—14 (43%)	6–10 series—2 (6%)
11–15 series—9 (27%)	11–15 series—0
> 15 series—1 (3%)	> 15 series—1 (3%)
*Males = 9 (100%)*	
0–5 series—3 (33%)	0–5 series—2 (22%)
6–10 series—3 (33%)	6–10 series—1 (11%)
> 11 series—0	> 11 series—0

When considering overall drug effectiveness alleviating symptoms and restoring normal functions of the musculoskeletal system, both females and males were not coherent in their responses. 10 out of 42 (24%) patients reported full restoration of their range of movements in the affected joint already after the first series of drug infusion. 2 of the patients (4.8%) did not feel any difference in the course of treatment and the other 2 patients (4.8%) had prior no such limitations. The mean number of series required to achieve the desired effect was 2.13 for males, and 3.1 for females. 1 patient (female) did not respond to the Pamidronate therapy and was withdrawn after 11 sets of drug infusion, while 2 different patients had to wait three times the median time (8 and 9 series) to notice their symptoms alleviate. The suggestion for the withdrawn patient was a treatment based on TNF-inhibitors.

Males required a mean of 1.63 series to mitigate pain, while females needed 2.72 series of drug infusion on average. However, the overall time required for total pain relief was significantly longer, averaging 3.38 series for males and 6 series for females. 1 patient (female) (2.4%) was in continuous pain, even after finishing the Pamidronate therapy.

**Fig. 1:**
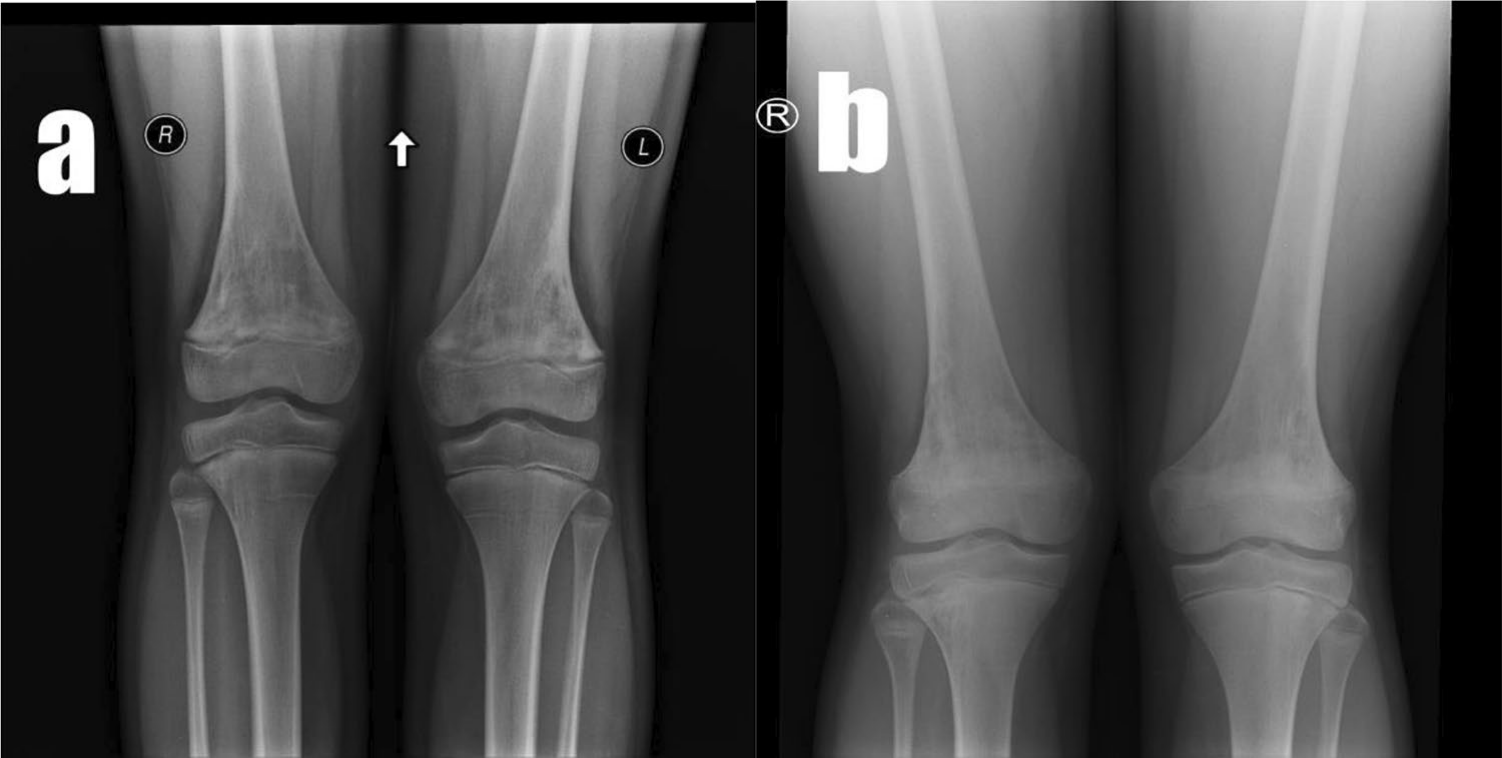
Distal femur bones lesions in the course of CRMO **a** before treatment **b** after treatment

An average of 2.75 series of Pamidronate was required to restore the diverted bone contours to normal shape in the male group (Fig. 1). Females however, observed the same results after an average of 4.41 series. In 4 cases (9.5%), no visible signs of swollen tissue were found prior to treatment.

### Safety of Pamidronate

**Fig. 2:**
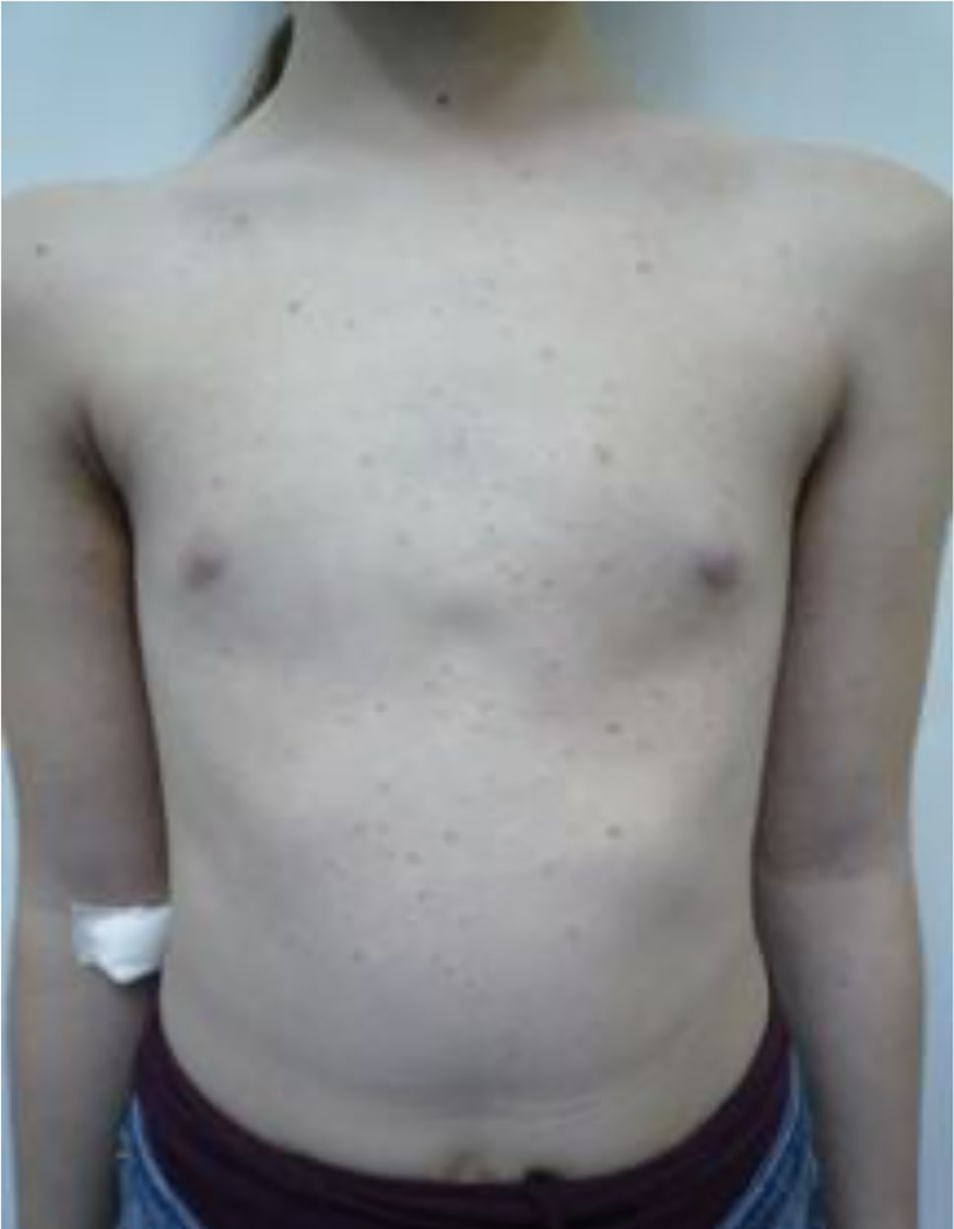
Skin rash developed during Pamidronate therapy (in color)

The total number of adverse drug reactions reported by patients was 105 (2.5 per patient). The most frequent adverse drug effect in the course of the therapy was episode of fever, which was indicated by 35 (83.3%) out of 42 respondents. 23 (55%) respondents had flu-like symptoms, 19 (45%) respondents reported visceral, abdominal pain and 18 (43%) patients reported lightheadedness. 20 patients (48%) confessed to having rigors and 8 out of 42 (19%) patients developed a skin rash or had some sort of an itching sensation (Fig. 2). Respondents also noted less frequently and occasional adverse drug reactions, such as: diarrhea (1), bone pain (4), joint pain (1), headache (3), vomitting (2), psychotic events and anxiety (1), hair loss (1), conjunctivitis (1). 10 patients (24%) indicated bone pain, headache, anxiety, fever, rash, rigors and hair loss as reactions that lasted for more than 3 days. One patient developed renal insufficiency after 15 sets of drug infusion. The overall adverse drug reaction distribution is presented in Table 2.

**Table 2:** Number of adverse drug reaction events regarding sex

Together = 42 (100%)	
0 side effects—3 (7%)	
1–2 side effects—12 (28.6%)	
3–4 side effects—13 (31%)	
5 and more side effects—14 (33.3%)	
Males = 9 (100%)	Females = 33 (100%)
0 side effects—0	0 side effects—3 (9%)
1–2 side effects—4 (44.4%)	1–2 side effects—8 (24%)
3–4 side effects—1 (11.1%)	3–4 side effects—12 (36%)
5 and more side effects—4 (44.4%)	5 and more side effects—10 (30%)

### Social and daily action influence of Pamidronate

20 patients (47.7%) were more willing to participate in social life and had better contact with their peers after the first infusion of Pamidronate. 10 patients (24%) did not feel any difference, while the remaining 13 (31%) denied it. 31 patients (71.5%) had a feeling of well-being following the first dose. The same number of patients 71.5% also agreed that they could forget about their discomfort between doses. 41 out of 43 patients (95%) shared the same opinion and would recommend Pamidronate therapy to cure CRMO disease.

Mobility assessment was based on the Childhood Health Assessment Questionnaire (CHAQ) and attempted to reveal if there were any differences in carrying out activities between following therapy steps. Taking into consideration every activity we asked about, we observed that after the third series of drug infusion at least three times more patients were able to complete the tasks without problems compared to the pre-treatment group. A more detailed description is provided in Table 3.

**Table 3:** Number of patients (percentage)

	Before implementing therapy		After first drug infusion series		After third drug infusion series	
*(a) Males daily activity during therapy*						
Ability to attend classes	0	2 (22.2%)	0	4 (44.4%)		
	1	2 (22.2%)	1	3 (33.3%)	0	9 (100%)
	2	3 (33.3%)	2	1 (11.1%)		
	3	2 (22.2%)	3	1 (11.1%)		
Ability to put on and carry backpack	0	3 (33.3%)	0	7 (77.7%)	0	9 (100%)
	1	3 (33.3%)	1	0 (0%)		
	2	1 (11.1%)	2	1 (11.1%)		
	3	2 (22.2%)	3	1 (11.1%)		
Ability to do favorite sport	0	1 (11.1%)	0	2 (22.2%)	0	7 (77.7%)
	1	2 (22.2%)	1	4 (44.4%)	1	1 (11.1%)
	2	2 (22.2%)	2	1 (11.1%)	2	0 (0%)
	3	4 (44.4%)	3	2 (22.2%)	3	1 (11.1%)
*(b) Females daily activity during therapy*						
Ability to attend classes	0	7 (21.2%)	0	11 (33.3%)	0	21 (63.6%)
	1	11 (33.3%)	1	12 (36.4%)	1	10 (30.3%)
	2	11 (33.3%)	2	8 (24.2%)	2	2 (6%)
	3	4 (12.1%)	3	2 (6%)	3	0 (0%)
Ability to put on and carry backpack	0	6 (18.2%)	0	11 (33.3%)	0	21 (63.6%)
	1	8 (24.2%)	1	8 (24.2%)	1	8 (24.2%)
	2	10 (30.3%)	2	10 (30.3%)	2	4 (12.1%)
	3	9 (27.2%)	3	4 (12.1%)	3	0 (0%)
Ability to do favorite sport	0	4 (12.1%)	0	6 (18.2%)	0	18 (33.3%)
	1	2 (6%)	1	9 (27.2%)	1	6 (24.2%)
	2	12 (36.4%)	2	7 (21.2%)	2	3 (30.3%)
	3	15 (45.5%)	3	11 (33.3%)	3	6 (12.1%)

0—perform without any limitations, 1—perform with little limitation, 2—perform with strong limitation, 3—cannot perform

## Discussion

Our study indicates that Pamidronate characterizes itself as an effective drug in alleviating symptoms of affected children. Moreover, the large number of adverse drug reaction events does not outweigh the impact it has on children’s social life and daily activities, thus it is of good repute. Needless to say, the authors truly believe that only a balanced combination of both doctor and patient perspectives is crucial for establishing a therapeutic alliance, which is necessary for successful treatment.

Lately, bisphosponates have obtained recognition as a first-line drug in the treatment of NSAIDs-refractory CRMO [17, 18]. The overall treatment of patients with CRMO is largely based on expert opinion supported by a relatively small number of cases. There is a lack of prospective clinical trials to determine the most appropriate drug and duration of treatment. In the currently published extensive data from a multi-center study based on the Eurofever international registry, it has been estimated that of 308 patients with CRMO, who had undergone second-line drug treatment, complete remission was the highest in the bisphosponates group (51%) [19]. The same study also noted that no response was observed in only 3% of patients from the aforementioned group [19]. This correlates with our results, in which 2% of patients were classified as non-responders. However, what is really captivating is the exact duration of treatment, it should elicit a specific response. It is generally accepted that Pamidronate treatment ought to last 9 months [20]. At the moment of filling out the survey, the mean duration of therapy was 20 months and it was still continued in 8 cases. In 2 cases, alleviation of symptoms was seen after 16 and 18 months of therapy, which is twice the time reported by Taddio et al. [20]. In order to achieve the desired effect, longer duration of the therapy may be required. Taking into account Pamidronate’s efficacy, Kerrison et al. [21] reported not only immediate relief of pain, but also improved activity and well-being in 7 children, who aborted NSAIDs. [21]

Other three authors Simm et al. [13], Miettunen et al. [14], Gleeson et al. [15] demonstrated both efficacy and safety measures of intravenous infusions of Pamidronate. Simm's results embraced pain reduction in 80% of patients after the first series of drug infusion, which compared with our 33% of patients’ pain alleviation may be likely overestimated due to the small population of the group. Miettunen’s study exhibited resolution of bone lesions on MRI in 90% of patients within six months of treatment. What Gleeson observed was not only pain relief in six out of seven children, but also regression of height loss caused by spinal fractures in response to therapy in 3 out of 5 children with affected vertebrae [15]. Adjacent soft tissue swelling resolution was noted by Compeyrot-Lacassagne et al. [22] within one week after Pamidronate. Our findings, however, are quite dissimilar, as only 4 out of 42 patients (10%) reported rapid swelling subsidence after the first series of Pamidronate. Furthermore, Miettunen et al. [14] stated that all his patients (*n* = 9) were able to attend classes within 1 week right after the first series of Pamidronate. Hence, in our study, we made an effort to delve into the issue and obtain more precise data regarding the level of difficulty of undertaken activities, such as attending school, the ability to carry a backpack or the ability to do a favorite sport. Compared to our results, only 15 out of 42 patients (36%) reported effortlessness, while another 15 patients (36%) indicated having mild difficulty in attending classes. In another multi-center survey conducted on patients affected by CRMO [23], between 25 and 61% of patients revealed a negative impact of CRMO on relationships, academic performance and also a negative impact on psychosocial well-being. In our study, 29 patients (69%) claimed they suspected a positive impact on the frame of mind following the first series of bisphosphonates.

It should be noted that the survey conducted by the authors presented a slightly different than so far described effectiveness of Pamidronate in treatment of CRMO. High efficiency of the therapy measured by clinical evaluation by physicians tended to be much better than the patient’s reported effectiveness. This fact can be caused either by smaller target population or by different perception of symptoms alleviation between those groups and may lead to drug effectiveness underestimation or overestimation. In our opinion, to control for different patient perceptions, clinical evaluation of Pamidronate effectiveness should be followed by a survey considering the patient’s opinion on the matter.

A great number of different chemical compounds and agents that comprise the bisphosphonates group have never been tested in a single randomized trial aiming to compare the effectiveness of CRMO symptoms’ resolution. While Pamidronic acid is believed to be the safest and the most effective one [13–15, 21], other drugs, such as Neridronic acid [18] or Zoledronic acid [24], have also proven themselves beneficial. Pamidronate’s safety profile was the key factor that determined our choice of this drug implementation. Recent recommendations do not clearly indicate a preferential second-line bisphosphonate treatment [11], but what remains interesting is a potential benefit of implementing them as a first-line treatment in patients with spinal involvement [25].

The demographic characteristics of our cohort do not differ significantly from those already described by other authors. The average onset of the disease occurs in around 14-year -old patients, which is slightly more than previously described [26–28]. In our case, females constitute a 3-time larger population than males, which resembles the general distribution of sex. [26–28].

The dosing regimen and schedule for Pamidronate was established empirically. The severity of the disease was taken into consideration when establishing the design and optimization of the dosage regimen. The final decision whether to discontinue therapy was based on both clinical evaluation and patients’ feedback.

Initially, some concerns have been expressed about safety and potential adverse effects caused by bisphosphonates. A heated debate has recently arisen on the long-term consequences of their effects on bones [29]. Moreover, Bisphosphonate-associated osteopetrosis once reported by Whyte et al. [30] may be nothing more than a coincidence, given the significant enhancement of bone mineral density shown by other authors [31–33]. However, between 2005 and 2010 alone, WHO counted more than 800 fragility fracture reports that could have been related to Bisphosphonates therapy [34–36]. In addition to the phenomenal value of Pamidronate in current orthopedics, this drug can often cause various adverse drug reactions, which may lead to patients’ non-compliance. The most common ones compiled by Robinson et al. [37] are fever and bone pain. While fever was reported by over 83% of our population, only approximately 10% of the patients mentioned having bone pain. This instant acute-phase reaction resulting in pyrexia, exhaustion or chills is thought to be caused by the release of pro-inflammatory cytokines, such as IL-6 and TNF-a [38]. Bisphosphonates are thought to be rarely the source of hives, or any type of rash [39]. In contrast to other studies, our research reveals a greater number of patients (9 out of 42—19%) complaining about their drug-related dermatological conditions. Renal insufficiency is rare, yet a very severe complication, which is proven to be associated with Pamidronate treatment [40]. A systematic review proposed by Tanvetyanon et al. [41] shows that acute renal failure occurred rather sporadically and usually after at least 11 months of continuous therapy. That is in line with our results, in which 1 patient developed renal impairment after 15 months of continuous treatment. Ocular complications, on rare occasions, may also be associated with infusion of Pamidronate leading to episcleritis, scleritis or transitory conjunctivitis [42], as was the case in our population. None of the patients were diagnosed with gastric lesions, as Pamidronate may alter restoration and preservation of gastric mucosal surface [43]. Two side effects of the drug that were not statistically confirmed in the available literature are worth mentioning as a possible consequence of the infusion of Pamidronate. These two adverse drug reactions are hair loss and psychotic events, which were described independently by two different patients.

Our study is not free of limitations. The authors recognize the possibility of participation bias, as not every selected patient was willing to participate in the online survey format. However, we were able to reach a larger sample more easily and managed to contact children scattered all over the country. To minimize recall bias, children were instructed to fill in the data under parental supervision. The data are self-reported, which may impact the accuracy of the information provided. Unfortunately, we were not able to eliminate reporting bias, as the nature of the study is descriptive and retrospective. In consequence, this fact precludes a unified research protocol; thus, these data should be treated with proper caution. Although performance was not perfect, by excluding open-ended questions, we focused to eliminate undesirable discrepancies between patients in reported data. No statistical analysis of the provided data was performed. Further investigation is warranted to investigate any changes in quantification of the quality of life in the course of Pamidronate therapy.

## Conclusion

NSAIDs-refractory CRMO affects children's social life and debilitates their daily school or sport routine. Pamidronate is considered very effective in restoring normal functions of the musculoskeletal system by patients; nevertheless, it is burdened with a high risk of adverse drug reactions. Although the noteworthy effectiveness at the end of the therapy was reported by 39 out of 42 (93%) respondents, more gradual onset of Pamidronate’s action was observed. The patient-reported effectiveness of Pamidronate differed in terms of symptoms alleviation between sexes. Patients delineated a larger than usual number of adverse drug reactions (2.5 per patient), including 8 cases (19%) of skin rash, which as described hiltherto should not have occurred so frequently. Only 3 patients (7%) were free from any ADRs. This research shows that measurement of Pamidronate efficacy in the treatment of CRMO should be followed by a self-reported and self-assessed survey of patients, because of discrepancies resulting from different perceptions of symptoms’ resolution between physicians and patients. The same self-reported and self-assessed survey should be considered while investigating number and distribution of adverse drug reactions of Pamidronate, as we showed more detailed descriptions provided by patients asked directly.

## Data Availability

The data and materials analyzed during current study are available from the corresponding author on reasonable request.
